# Efficacy and safety of Cerebrolysin treatment in early recovery after acute ischemic stroke: a randomized, placebo-controlled, double-blinded, multicenter clinical trial

**Published:** 2017

**Authors:** K Gharagozli, AA Harandi, S Houshmand, N Akbari, DF Muresanu, J Vester, S Winter, H Moessler

**Affiliations:** * Department of Neurology, Shahid Beheshti Medical University, Teheran, Islamic Republic of Iran; ** Department of Clinical Neurosciences, “Iuliu Hatieganu” University of Medicine and Pharmacy, Cluj-Napoca, Romania; *** RoNeuro Institute for Neurological Research and Diagnostic, Cluj-Napoca, Romania; **** Department of Biometry and Clinical Research, IDV Data Analysis and Study Planning, Krailling, Germany; ***** EVER Neuro Pharma GmbH, Unterach, Austria; ****** COMAMO Lifesciences GmbH, Mondsee, Austria

**Keywords:** ischemic stroke, rehabilitation, RCT, Cerebrolysin, early recovery

## Abstract

***Background and Purpose***:

The aim of this study was to evaluate the efficacy, safety, and tolerability of cerebrolysin in the early recovery phase after acute ischemic stroke.

**Methods.** This prospective, randomized, double-blinded, placebo-controlled, multicenter, parallel-group study enrolled a total of 100 patients within 18 h after the onset of stroke. The patients were treated with Cerebrolysin (30 mL over seven days followed by 10 mL until day 30) or placebo once daily over a period of four weeks. Efficacy was primarily assessed by the NIH Stroke Scale at day 30, and additional parameters included the modified Rankin Scale, the Clinical Global Impression, the Patient Global Satisfaction (PGS) and the Mini Mental State Examination (MMSE). Nonparametric statistical procedures employing the Wilcoxon-Mann-Whitney test were used for data analysis. Safety and tolerability were assessed by adverse events, vital signs, and laboratory parameters.

**Results.**The estimated effect size on the change from baseline in the NIH Stroke Scale on day 30 indicated a medium to large superiority of cerebrolysin compared to placebo (Mann-Whitney [MW] 0.66; 95% confidence interval [CI] 0.55-0.78, P=0.005). Similar effect sizes were reported for the modified Ranking Scale (MW 0.65; 95% CI 0.54-0.76; P=0.010) and the Clinical Global Impression (MW 0.70; 95% CI 0.55-0.85; P=0.006). Effect sizes in the MMSE and PGS did not reach statistical significance. No significant group differences were seen in any of the safety parameters.

**Conclusions.** Cerebrolysin was effective, safe, and well tolerated in the early recovery phase after acute ischemic stroke and significantly improved neurological and global function outcomes compared to placebo.

## Introduction

The recovery process from stroke is complex. Pharmacological treatment options after acute care are scarce, and even modest benefits may be of high relevance for the patients. Most clinical trials to date were designed without consideration of the complexity of the progression of ischemic damage, in which a series of noxious physiological, biochemical and molecular events interact in a time-dependent sequence. Consequently, practically all clinical trials for neuroprotective agents that target single events in the ischemic cascade have failed so far [**1**], and it has been proposed that multimodal drugs targeting several steps in the ischemic cascade might be more successful. In particular, agents that, in addition to their neuroprotective properties, stimulate and enhance neuroplasticity during the recovery process are believed to hold the greatest promise. 

Cerebrolysin is a neuropeptide preparation with neuroprotective and neuroplastic properties similar to endogenous neurotrophic factors. These pharmacologic effects are of relevance for cerebrovascular and neurodegenerative diseases, as these diseases generate a pathological environment, which is deleterious for neurons, causing their degeneration, dysfunction and death either immediately or over time. Experimental studies in stroke models have shown that Cerebrolysin stabilizes the structural integrity of neurons via the inhibition of calpain, supports the formation of neuronal networks by inducing neuronal sprouting and neurogenesis, and enhances the functional recovery accompanied by a reduction in infarct volume [**2**-**4**]. The molecular basis for increased neurogenesis and improved functional performance after the administration of Cerebrolysin was found in the modulation of the phosphatidylinositol-3-kinase (PI3)/AKT, glycogen synthase kinase 3 beta (GSK3β) and Sonic hedgehog (Shh) signaling pathways, the latter being currently discussed as a pivotal pathway in modulating blood-brain barrier integrity and neuroprotection post-stroke [**5**-**7**]. 

Previous clinical research on Cerebrolysin was mainly performed in an acute stroke setting with daily drug administration by intravenous infusion, usually of 30 mL for 10 d [**8**-**10**]. Recent randomized, controlled studies have been performed in stroke rehabilitation with a treatment duration of 21 d and concomitant participation in a rehabilitation program [**11**,**12**]. These studies reported significant early treatment effects of Cerebrolysin on neurological functions and a significant treatment effect on the recovery of motor functions in the upper limbs, especially in more severely affected stroke patients.

Based on these results, a randomized, placebo-controlled, double-blinded study was designed to investigate the clinical efficacy, safety, and tolerability of a four-week treatment with Cerebrolysin during the early recovery period after ischemic stroke.

## Methods

***Study Design and Treatment Regimen***

This prospective, randomized, double-blinded, placebo-controlled, multicenter, parallel-group study evaluated the safety and efficacy of Cerebrolysin in the early recovery period after acute ischemic stroke. All patients received the study medication as an add-on to standard therapy, which consisted of 100 mg daily aspirin, pentoxifylline or low-dose heparin. The first application of the study medication was within 18 h post-stroke, and the treatment lasted for four weeks. During the first seven days, the study drug was administered as intravenous infusion for over 30 minutes. Placebo treatment consisted of 80 mL physiological saline (0.9% sodium chloride). Cerebrolysin (30 mL) was diluted with physiological saline to a total volume of 80 mL. In the post-acute phase, a daily dosage of 10 mL of the study medication was administered by slow intravenous injection on the first five days of each week. This prolonged treatment regimen was chosen based on previous study results and in order to support the longer-lasting neuronal recovery processes in the brain [**8**,**9**,**11**,**13**,**14**]. 

Three neurological hospitals in the Islamic Republic of Iran participated in this study. All procedures performed in studies involving human participants were in accordance with the ethical standards of the institutional and/ or national research committee and with the 1964 Helsinki declaration and its later amendments or comparable ethical standards.

***Inclusion and Exclusion Criteria***

This study included patients between 45 and 85 years old with a clinically confirmed acute embolic or thrombotic stroke in the territory of the arterial branches of the internal carotid artery. 

Patients were excluded for the following reasons: complete remission of symptoms within 4 h after onset, the presence of signs and symptoms of progressive neurological deficits, signs of hemorrhagic stroke or intracranial bleeding, systolic blood pressure ≥ 200 mm/ Hg, diastolic blood pressure ≥ 100 mm/ Hg, signs of stupor or coma (Glasgow Coma Scale [GCS] score [**15**] of ≤ 6), convulsions, pupillary edema, increased intracranial pressure, myocardial infarction, cardiac function deficit, renal or hepatic insufficiency, acute infections, pregnancy, or participation in another clinical study. Patients treated with recombinant tissue plasminogen activator (rtPA) were not included in the study, and concomitant medication with piracetam or calcium channel blockers was not allowed due to their purported neuroprotective characteristics.

***Randomization and Blinding***

Patients were assigned to treatment groups according to a predefined randomization plan by using a block size of 4, a ratio of 1:1, and stratified by study center. Patients, investigators and all study personnel were blinded to the treatment allocation. The statistician in charge of randomization was unblinded but was not involved in any other study-related procedures. EVER Neuro Pharma provided the study medication in 10-mL ampoules labeled appropriately to maintain blinding. 

***Efficacy and Safety Criteria***

The efficacy of Cerebrolysin was assessed by the changes from baseline in the National Institutes of Health Stroke Scale (NIHSS) [**16**], the modified Rankin Scale (mRS) [**17**-**19**], the Clinical Global Impression (CGI) [**20**], the Patient Global Satisfaction (PGS; a hospital specific patient self-rating scale), and the Mini Mental State Examination (MMSE) [**21**]. Efficacy assessments were performed at baseline and at days 1, 3, 7, 14, and 30 post-stroke. 

Safety was assessed by adverse events, vital signs, and laboratory parameters. Vital signs, including the level of consciousness, were closely monitored within the first week. Laboratory parameters, including a complete blood count, were assessed at baseline, day 7 and day 30.

Statistical Analysis

The primary objective of this trial was to investigate the hypothesis that patients randomized to receive Cerebrolysin treatment would show greater improvement from baseline in the NIHSS scores over the 30th day of the study compared to those randomized to receive placebo. The level of statistical significance was set to α=0.05 (two-sided test for superiority). As all outcome scales were either ordinal or rating scales with skewed distributions, the robust nonparametric Wilcoxon-Mann-Whitney test [**22**-**25**] was chosen in order to minimize assumptions [**26**]. The corresponding effect size measure was the Mann-Whitney (MW) estimator, which indicated the probability that a patient treated with the test treatment achieved a better result than when treated with the control treatment. The MW ranged from 0 to 1, with 0.5 indicating equality and statistically defined as P (X<Y) + 0.5 P (X=Y). The relevant benchmark values for the MW were 0.29 (large inferiority), 0.36 (medium inferiority), 0.44 (small inferiority), 0.50 (equality), 0.56 (small superiority), 0.64 (medium superiority) and 0.71 (large superiority) [**27**,**28**].

All analyses were performed on a modified Intention-to-Treat (mITT) analysis data set by using the last-observation-carried-forward (LOCF) approach for the handling of missing data. The mITT analysis set was defined as all randomized patients who received at least one dose of study medication and provided at least baseline and one post-baseline assessment for the respective outcome measure. The mITT dataset using the data as an available approach (observed cases; OC) was analyzed to check for the robustness of the primary mITT-LOCF analysis. Due to baseline inhomogeneity in the NIHSS and mRS scores, an analysis of covariance (ANCOVA) was performed as a sensitivity analysis using the respective baseline score as a covariate. Baseline anamnestic characteristics were analyzed and compared to 2x2 tables and Fischer’s exact test. A repeated-measures analysis of variance (ANOVA) was used to analyze changes in the laboratory parameters over time and between groups. In addition to these statistical tests, classic and robust summary statistics were calculated for each variable.

Due to the exploratory nature of this study, a formal sample size calculation was not conducted; however, a sample size of approximately 100 patients was deemed appropriate.

## Results

***Study Population***

A total of 100 patients were enrolled in this study (Cerebrolysin, n=50; placebo, N=50). All patients received at least one dose of the study medication and thus represented the safety analysis data set. One patient in the Cerebrolysin group had no efficacy baseline assessment, and another patient in the Cerebrolysin group did not provide any post-baseline efficacy data. Thus, the mITT-LOCF analysis consisted of 98 patients (Cerebrolysin, N=48; placebo, N=50). Of those, 23 patients discontinued participation in the study prematurely: four patients withdrew because of adverse events (AEs) (Cerebrolysin, N=2; placebo, N=2), three patients died in the acute phase due to stroke severity (Cerebrolysin, N=1; placebo, N=2) and another 16 patients were lost to follow-up after discharge from the hospital (Cerebrolysin, N=12; placebo, N=4). Thus, a total of 75 patients (Cerebrolysin, N=33; placebo, N=42) comprised the data as the available (observed cases; OC) analysis set. Since the percentage of missing values at day 30 was comparatively high (23%), a sensitivity analysis for the primary outcome measure was also performed based on the OC population. 

The mean age of the patients was 68 years, and 53% were male. The patients had no significant pre-stroke disability and an NIHSS score of 4-24, both inclusive. The medical history and risk factors did not show relevant group differences, except for obesity, which was more frequent in the placebo group (p=0.01), and aphasia, which was significantly more frequent in the Cerebrolysin group (p=0.04; **Table 1**).

The baseline scores for the efficacy parameter revealed some inhomogeneity between the two treatment groups (**Table 1**). Overall, patients in the placebo groups presented with somewhat milder symptoms according to the NIHSS score (9.1± 4.8; mean ± standard deviation) compared to the Cerebrolysin group (11.1 ± 5.0). Corresponding evidence for a slightly milder patient population in the placebo group was also seen in the mRS score (placebo: 3.4 ± 1.1; Cerebrolysin: 3.9 ± 1.0). Since these baseline group differences reached statistical significance for the NIHSS (MW 0.36, P=0.02) and for the mRS (MW 0.39, P=0.05), an ANCOVA sensitivity analysis was performed by using the baseline score as a covariate to confirm the robustness of the data. 

**Table 1:** Comparison of baseline characteristics and risk factors (safety set)

Baseline characteristics	N (Cere/ Pla)	Cerebrolysin	Placebo	p-value	
Sex - Male (N, %)	50/50	27 (54%)	26 (52%)	ns	
Age (Mean, SD)	49/49	69.0 (10.7)	66.5 (12.2)	ns	
NIHSS Score (Mean, SD)	49/50	11.1 (5.0)	9.1 (4.8)	0.02a	
mRS Score (Mean, SD)	47/49	3.9 (1.0)	3.4 (1.1)	0.05b	
Prevalence of risk factors	N (Cere/ Pla)	Cerebrolysin	Placebo	OR	p-valuec
Previous Stroke	50/50	28%	18%	1.8	ns
Weakness	50/50	24%	22%	1.1	ns
Aphasia	50/50	28%	10%	2.8	0.04
Cardiac Disease	50/50	28%	36%	0.8	ns
Past/ Current Smoker	50/50	22%	28%	0.8	ns
Hypertension	50/50	78%	82%	1.0	ns
Obesity	50/50	28%	56%	0.5	0.01
Increased Serum Lipids	50/50	56%	62%	0.9	ns
Diabetes	50/50	38%	34%	1.1	ns
Family History of Stroke	50/50	20%	8%	2.5	ns
Cere = Cerebrolysin; NIHSS = National Institutes of Health Stroke Scale; mRS = modified Rankin Scale; ns = not significant; OR = odds ratio; Pla = placebo; SD = standard deviation. aWilcoxon-Mann-Whitney test (MW=0.36); bWilcoxon-Mann-Whitney test (MW=0.39); cFisher’s exact test; data as available.					

***Primary Efficacy Analysis (NIHSS)***

**Fig. 1:**
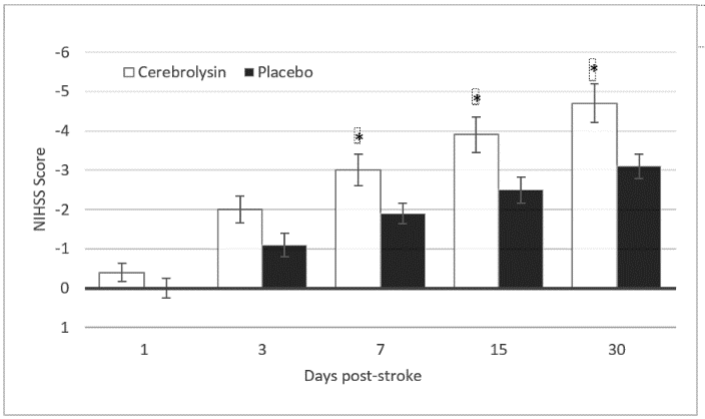
Changes from baseline in the NIHSS for Cerebrolysin (30 mL/ d) and placebo over the 30th day. The values represent the arithmetic mean ± standard error of the mean. Assessments were performed at 1, 3, 7, 15, and 30 days post-stroke. The mITT-LOCF population included 98 patients (Cerebrolysin, N=48; placebo, N=50)

The mean NIHSS scores decreased from 11.1 ± 5.0 at baseline to 6.2 ± 5.1 on day 30 in the Cerebrolysin group and from 9.1 ± 4.8 to 6.0 ± 4.8 in the placebo group (**Fig. 1**). The mean changes in the NIHSS scores at 30 d post-stroke compared to those at baseline were -4.7 ± 3.4 for the Cerebrolysin group versus -3.1 ± 2.2 for the placebo group. The time-course revealed a constant increase in the effect size, which peaked on day 30 (**Fig. 1**). 

The time-course of the OC population was similar to the results of the primary LOCF analysis, with a final mean NIHSS score of 6.0 ± 5.1 for the placebo group and 6.1 ± 5.2 for the Cerebrolysin group. Thus, the handling of the missing data had a negligible impact on the results, which attests the robustness of the study results. The change from baseline to day 30 post-stroke was slightly greater for the OC population for both groups compared to the LOCF dataset (Cerebrolysin: -5.3 ± 3.1; placebo: -3.3 ± 2.3), confirming that the LOCF methodology for the handling of the missing data represented the more conservative approach with respect to the observed effect size. 

**Fig. 2:**
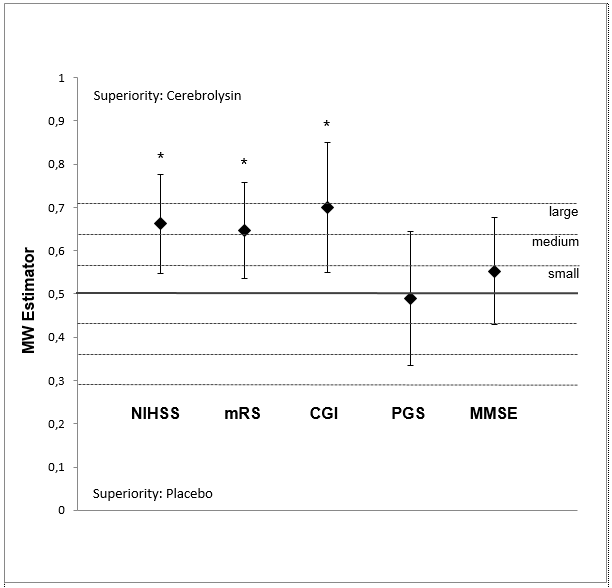
Changes from baseline in the outcome scales at day 30 for Cerebrolysin (30 mL/ d) and placebo. The values represent the Mann-Whitney (MW) effect sizes (95% CI; Wilcoxon-Mann-Whitney test). In the mITT-LOCF, population data were available from 98 patients (Cerebrolysin, N=48; placebo, N=50) for the NIHSS, from 96 patients (Cerebrolysin, N=47; placebo, N=49) in the mRS, from 60 patients (Cerebrolysin, N=24; placebo, N=36) in the CGI, from 54 patients (Cerebrolysin, N=19; placebo, N=35) in the PGS and from 78 patients (Cerebrolysin, N=34; placebo, N=44) in the MMSE. *p<0.05, Wilcoxon-Mann-Whitney test. CGI = Clinical Global Impression; MMSE = Mini-Mental State Examination; mRS = modified Rankin Scale; NIHSS = National Institutes of Health Stroke Scale; PGS = Patient Global Satisfaction

The nonparametric mITT-LOCF analysis demonstrated a highly significant, medium to large superiority of Cerebrolysin relative to placebo from day 7 onwards, peaking at day 30, with an MW equal to 0.66 (95% CI: 0.55-0.78; P=0.005; **Fig. 2**). A sensitivity analysis by means of analysis of covariance (ANCOVA) with the baseline score as a covariate confirmed the results of the nonparametric analysis (PAncova = 0.0249; two-sided) and attested the robustness of the nonparametric analysis. 

***Secondary Efficacy Criteria***

**Fig. 3:**
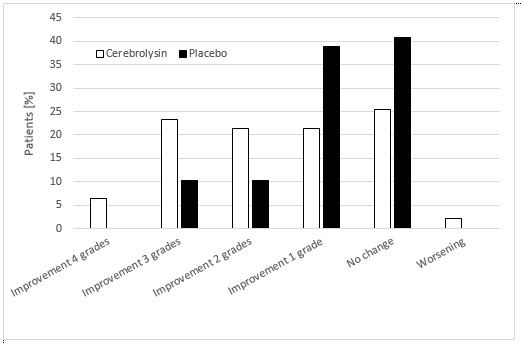
Changes from baseline in the modified Rankin Scale (mRS) for Cerebrolysin (30 mL/ d) and placebo over the 30th day. The values represent the percentage of patients showing improvement, no change or worsening. For the mRS, the mITT-LOCF population provided data from 96 patients (Cerebrolysin, N=47; placebo, N=49)

Similar to the results for the NIHSS, substantial differences were found between the Cerebrolysin and placebo groups at day 30 for both the mRS and the CGI. At day 30, 24 Cerebrolysin-treated patients (51.1%) experienced a substantial improvement from baseline of ≥ 2 points in the mRS versus only 10 patients (20.4%) from the placebo group (**Fig. 3**). An excellent outcome (mRS 0 to 1) was reported for 31.3% of the Cerebrolysin patients versus 26.0% of the placebo group. The median change from baseline to day 30 for the mRS was -1.0 point for placebo versus -2.0 for the Cerebrolysin group, and the nonparametric analysis (**Fig. 2**) demonstrated a significant, medium to large superiority of Cerebrolysin relative to placebo, with an MW equal to 0.65 (95% CI: 0.54-0.76; P=0.01). A sensitivity ANCOVA analysis with the baseline score as a covariate (PAncova = 0.0082; two-sided) confirmed this finding. 

Similar results were observed in the CGI at day 30. While patients on placebo did not improve in the CGI (0.3 ± 1.2), patients on Cerebrolysin improved by -0.9 ± 1.8 points. The nonparametric analysis showed a large and highly significant superiority of Cerebrolysin (**Fig. 2**), with an MW equal to 0.7 (95% CI: 0.55-0.85; p=0.006). 

In both the MMSE and the PGS, improvements were higher in the Cerebrolysin group compared to placebo (MMSE, Cerebrolysin: 1.7 ± 2.9 points, placebo: 1.0 ± 1.4 points; PGS, Cerebrolysin: 16.8 ± 27.4, placebo: 13.0 ± 17.5); however, the differences did not reach statistical significance.

***Safety and Tolerability***

Of the patients treated with Cerebrolysin, 24% (N=12) reported at least one adverse event (AE) compared to 28% (N=14) of the placebo patients (**Table 2**). AEs were rated as mild in severity and not related to study medication. In each study group, two patients suffered from seizures; these serious adverse events (SAEs) led to the withdrawal from the study. Three patients died due to the severity of the stroke: one in the Cerebrolysin group and two in the placebo group.

**Table 2:** Reported adverse events

Reported Term	Cerebrolysin N=50	Placebo N=50
Nausea	3	1
Vomiting	4	1
Headache	4	3
Confusion	1	5
Vertigo	3	1
Insomnia	1	0
Anorexia	2	2
Seizures	2	2
AEs related to study drug	0	0
AEs leading to withdrawal	2	2
Patients with AEs (N, %)	12 (24%)	14 (28%)
Of these, patients with SAEs (N, %)	2 (4%)	2 (4%)
Death (N, %)	1 (2%)	2 (4%)

Vital signs were similar between the treatment groups, and the heart rate, body temperature and respiratory rate did not show any clinically relevant changes during the course of the study in either group. The systolic and diastolic blood pressure declined in the first week after the stroke in both groups, probably because of the standard stroke care. No relevant differences in the level consciousness (GCS) were noted between the groups, and both groups showed improved GCS scores over time. The laboratory values were measured on days 1, 7, and 30 and did not exhibit any significant or clinically relevant differences between the treatment groups. Furthermore, no trends towards specific pathological laboratory findings were detected in any of the groups.

Overall, the safety outcome reflected the expected safety and tolerability profile of Cerebrolysin in patients after acute ischemic stroke.

## Discussion

This randomized, placebo-controlled, double-blinded study has shown a medium to large superiority of Cerebrolysin versus placebo in the NIHSS, the primary outcome parameter assessing the recovery of neurological functions early after stroke, at day 30. A similar effect size of Cerebrolysin has also been reported for the mRS and the CGI. The improvements of neurological deficits and global functions were robust and of high clinical relevance. Small improvements were observed in the cognitive domain, as evidenced by the MMSE; however, the MMSE has limitations in acute stroke, especially due to its low sensitivity, particularly to impairments in abstract reasoning, executive functioning, and visual perception/ construction [**29**]. Furthermore, the assessment of cognitive changes 30 days post-stroke may have been conducted too early.

A comparison of the patient population at baseline indicated a significant difference in stroke severity, with patients in the Cerebrolysin group scoring two points worse in the NIHSS and 0.5 points worse in the mRS compared to patients in the placebo group. Possible bias due to this baseline imbalance was excluded by sensitivity analyses of the NIHSS and mRS score at the study endpoint by ANCOVA using the baseline value as a covariate. Furthermore, the primary non-parametric analysis implicitly corrected for these baseline differences, as it analyzed the score change from baseline to the study endpoint and not the absolute scores. The results of these sensitivity analyses were fully in line with main analyses and confirmed the statistically significant superiority of Cerebrolysin. This clearly attested the robustness and confirmed the validity of our results. 

The rate of premature discontinuation due to adverse events and mortality was relatively low and balanced between the groups; however, the number of patients lost to follow-up for administrative reasons at day 30 was relatively high, especially due to patients living far from the study center. To determine whether the missing values had any impact on the study results, we conducted a sensitivity analysis using the data as an available approach in addition to a primary LOCF analysis. The effect sizes of all outcome parameters were nearly identical for both datasets, indicating that the missing data had no impact on the study results.

The primary outcome in the majority of clinical stroke studies was the mRS at day 90, since it is commonly believed that the recovery after stroke reaches its peak at day 90. While this approach is widely accepted among stroke trialists, it has certain shortcomings. In particular, the inability to fully control confounding factors in the relatively long study period after the acute hospital care, such as differences in the type and intensity of neurorehabilitation programs, differences in the family setting and level of home care, or unrelated adverse events, which clearly impact the outcome after stroke and introduce significant variability and, potentially, bias. The mRS, a relatively crude global measure of improvement, which is influenced by a variety of factors that stroke treatment may not influence, might be especially prone to such bias. Consequently, stroke trials focusing on a day 90 global outcome require large patient numbers, as well as sophisticated study designs to minimize the potential bias. We used the NIHSS at day 30 as the primary outcome parameter to measure the recovery of neurological function to cope with these shortcomings. The NIHSS is a very sensitive and valid measure of treatment effects in the early recovery period after stroke up to day 30 [**30**,**31**]. Importantly, previous research has also shown that the NIHSS is a good predictor of long-term stroke outcome [**32**,**33**].

Based on these considerations, we expect that the beneficial treatment effect that we have observed at day 30 are a valid predictor of the long-term neurological outcome of the patients in our trial. 

The results of the current study are in line with previous findings, showing an early and significantly accelerated recovery after stroke following to treatment with Cerebrolysin [**8**-**10**,**12**-**14**,**34**-**36**]. Furthermore, it appeared from previous study results that patients who were more severely affected at baseline tended to respond better to Cerebrolysin treatment, with less severe stroke deficits [**37**] or greater improvement in motor functions [**11**,**38**] at study endpoint. A subgroup analysis of the CASTA trial [**9**] showed that patients with a NIHSS of >12 (n=246) at baseline improved by three points more in the NIHSS at day 90 than patients in the placebo group, while in the overall population, improvement was less pronounced. Similarly, a subgroup analysis of patients with more severe motor impairment at baseline (Fugl-Meyer Assessment < 50) showed a significant improvement in motor function versus placebo in the ECOMPASS trial [**12**]. Again, the results of our study were in line with the previous findings, as we have demonstrated significant improvement in the NIHSS in a more severely affected patient population (mean NIHSS at baseline: Cerebrolysin, 11.1 ± 5.0; placebo, 9.1 ± 4.8). It is of special interest that the significant improvement in neurological functions in our study was observed even without a standardized concomitant rehabilitation program. Active exposure to a rehabilitation intervention in addition to the pharmacological intervention may have provided a further clinical benefit. Such a synergistic effect of Cerebrolysin and a standardized rehabilitation program has recently been demonstrated in a larger clinical trial [**11**].

As expected from previous research and clinical experience, Cerebrolysin was well tolerated, and there were no safety issues or unexpected findings in any of the safety parameters. 

In conclusion, our results confirm and extend the findings of previous studies and clearly attest to positive benefit-risk ratio of Cerebrolysin treatment in acute stroke. The relatively low patient number in our study might have limited the degree of evidence; however, the results of our study are robust and in line with the previous trials. 

**Acknowledgements**

EVER Neuro Pharma GmbH provided the study medication.

**Conflict of Interest**

JV is a member of the advisory board of EVER Neuro Pharma. SW is an employee of EVER Neuro Pharma. HM is a scientific consultant for EVER Neuro Pharma. The other authors declare that they have no conflict of interest.
